# Complete Genome Sequence of *Amycolatopsis* sp. CA-230715, Encoding a 35-Module Type I Polyketide Synthase

**DOI:** 10.1128/MRA.00805-21

**Published:** 2021-09-23

**Authors:** Tue S. Jørgensen, Tetiana Gren, Daniel Oves-Costales, Francisco Javier Ortiz-López, Daniel Carretero-Molina, Ignacio González, Kai Blin, Olga Genilloud, Tilmann Weber

**Affiliations:** a The Novo Nordisk Foundation Center for Biosustainability, Technical University of Denmark, Kongens Lyngby, Denmark; b Fundación MEDINA, Centro de Excelencia en Investigación de Medicamentos Innovadores en Andalucía, Granada, Spain; Montana State University

## Abstract

We report the sequencing, assembly, and annotation of the genome of *Amycolatopsis* sp. CA-230715, a potentially interesting producer of natural products. The genome of CA-230715 was sequenced using PacBio, Illumina, and Nanopore technologies. It consists of a circular 10,363,158-nucleotide (nt) chromosome and a circular 12,080-nt plasmid.

## ANNOUNCEMENT

The genus *Amycolatopsis* is a recognized source of secondary metabolites (1–3). Only a few complete gapless genome sequences from the *Amycolatopsis* genus exist in public databases. Here, we report the sequencing of *Amycolatopsis* sp. CA-230715, which was identified in antimicrobial screening against Acinetobacter baumannii MB5973. The strain was isolated from a soil sample collected in Berbérati (Central African Republic). The original colony was isolated from a serial dilution of a soil suspension plated onto HANOB medium (6.25 g/liter NaNO_3_, 2.5 g/liter K_2_HP_4_, 0.65 g/liter MgSO_4_, 1.25 g/liter humic acid, 0.020 g/liter benomyl, pH 7) after incubation for 5 weeks at 28°C/70% relative humidity. For DNA isolation, the strain was grown in liquid yeast extract-malt extract (YEME) medium (4). DNA was isolated using the Genomic-tip G100 kit (Qiagen, Venlo, Netherlands). PacBio RS II (Pacific Biosciences, Menlo Park, CA, USA) data were generated by Macrogen Inc. (Seoul, South Korea; DNA/polymerase binding kit P6; SMRT cell 8Pac v3 using g-TUBE-sheared and Blue Pippin-size-selected DNA), yielding 125,283 subreads with an *N*_50_ value of 16,071 nucleotides (nt). Subread generation and adapter removal were performed using SMRT Analysis v2.3 software. A KAPA HyperPlus library was sequenced on an Illumina MiSeq instrument (San Diego, CA, USA), yielding 4,477,879 read clusters (2 × 150 nt), totaling 1,273,625,221 nt. Nanopore data were generated on a MinION device using the SQK-RBK004 kit and a FLO-MIN106D R9.4 Rev-D flow cell (183,945 reads; *N*_50_, 10,904 nt; total, 1,065,369,647 nt) (Oxford Nanopore Technologies, Oxford, UK). Default software parameters were used except where otherwise noted. The Illumina reads were adapter and quality trimmed using AdapterRemoval2 v2.1.7 (5) with the parameters --trimns --trimqualities. The Nanopore reads were demultiplexed and base called using Guppy v3.0.3, adapter trimmed using Porechop v0.2.4 (6), and assembled using Flye v2.4.1-geb89c9e (7). This assembly was then polished with the Illumina reads using the polishing module in Unicycler v0.4.7 (8) and used in a second round of assembly with Unicycler v0.4.7 (running SPAdes v3.13.0) (9), which combined the initial polished assembly with the Illumina and PacBio data sets. The contiguity and circular topology of the 10,363,158-nt chromosome (GC content, 69.7%) and circular 12,080-nt plasmid (GC content, 67.6%) were evaluated using the assembly graph from Unicycler and Bandage v0.8.1 (10). The circular chromosome was not rotated. The assembly had a BUSCO v3.1.0 (actinobacteria_odb9) (11) score of 100% complete genes (352 genes; 5 in duplicate). The genome sequence was annotated using Prokka v1.14.0 (12) with additional databases as described in reference 13. A total of 9 rRNAs and 104 tRNAs were found in the annotation, as well as 9,465 coding DNA sequences (CDS), of which 6,405 (68%) were functionally annotated.

According to antiSMASH v6.0.0 (14), the strain harbors potentially novel biosynthetic gene clusters (BGCs), including a 35-module, 216-kb type I polyketide synthase BGC, one of the largest uninterrupted bacterial BGCs reported.

AutoMLST analysis (15) of the genome sequence has shown that *Amycolatopsis* sp. CA-230715 has 82.1% average nucleotide identity (ANI) similarity to Amycolatopsis nigrescens CSC17Ta-90, and GTDB-tk v1.5.1, R202 (16), places the strain within the *Amycolatopsis* genus.

### Data availability.

All data are available under BioProject accession number PRJNA639419. The raw reads have been deposited at the SRA under accession numbers SRR12367306 (Illumina), SRR12367305 (PacBio), and SRR12367307 (Nanopore). The GenBank accession numbers are CP059997.1 (chromosome) and CP059998.1 (plasmid).
